# Anti-Sigma Factors in *E. coli*: Common Regulatory Mechanisms Controlling Sigma Factors Availability

**DOI:** 10.2174/1389202911314060007

**Published:** 2013-09

**Authors:** Luis Gerardo Treviño-Quintanilla, Julio Augusto Freyre-González, Irma Martínez-Flores

**Affiliations:** 1 Departamento de Tecnología Ambiental, Universidad Politécnica del Estado de Morelos. Blvd. Cuauhnáhuac 566, Col. Lomas del Texcal, 62550. Jiutepec, Morelos, México;; 2 Programa de Genómica Evolutiva, Centro de Ciencias Genómicas, Universidad Nacional Autónoma de México. Av. Universidad s/n, Col. Chamilpa, 62210. Cuernavaca, Morelos, México;; 3 Departamento de Genómica Computacional, Centro de Ciencias Genómicas, Universidad Nacional Autónoma de México. Apdo. Postal 510-3, 62250. Cuernavaca, Morelos, México

**Keywords:** Anti-sigma factors, *Escherichia coli*, Homeostasis, Negative feedback, Sigma factors, Transcriptional regulation.

## Abstract

In bacteria, transcriptional regulation is a key step in cellular gene expression. All bacteria contain a core RNA polymerase that is catalytically competent but requires an additional σ factor for specific promoter recognition and correct transcriptional initiation. The RNAP core is not able to selectively bind to a given σ factor. In contrast, different σ factors have different affinities for the RNAP core. As a consequence, the concentration of alternate σ factors requires strict regulation in order to properly control the delicate interplay among them, which favors the competence for the RNAP core. This control is archived by different σ/anti-σ controlling mechanisms that shape complex regulatory networks and cascades, and enable the response to sudden environmental cues, whose global understanding is a current challenge for systems biology. Although there have been a number of excellent studies on each of these σ/anti-σ post-transcriptional regulatory systems, no comprehensive comparison of these mechanisms in a single model organism has been conducted. Here, we survey all these systems in *E. coli* dissecting and analyzing their inner workings and highlightin their differences. Then, following an integral approach, we identify their commonalities and outline some of the principles exploited by the cell to effectively and globally reprogram the transcriptional machinery. These principles provide guidelines for developing biological synthetic circuits enabling an efficient and robust response to sudden stimuli.

## INTRODUCTION

In bacteria, the regulation of gene expression is the basis for adaptability, morphogenesis, and cellular differentiation. From all the different regulatory layers, regulation of transcription initiation is a very important step for controlling gene expression. All eubacteria contain a single ∼380-kDa multi-subunit α_2_ββ´ω RNA polymerase (RNAP) core enzyme, which is catalytically competent and able to recognize DNA non-specifically. However, it requires an additional dissociable σ factor for specific promoter recognition and correct transcription initiation. Most σ factors belong to the σ^70^family, whose members contain two domains with a high degree of sequence and structural conservation: 1) σ2, which binds the RNAP β′ subunit coiled-coil and the -10 promoter element, and 2) σ4, which binds the RNAP β subunit flap and -35 promoter elements. An additional σ family, σ^54^, does not share any significant sequence similarity with the σ^70^ family and is functionally distinct [1].

**Fig. (1):**
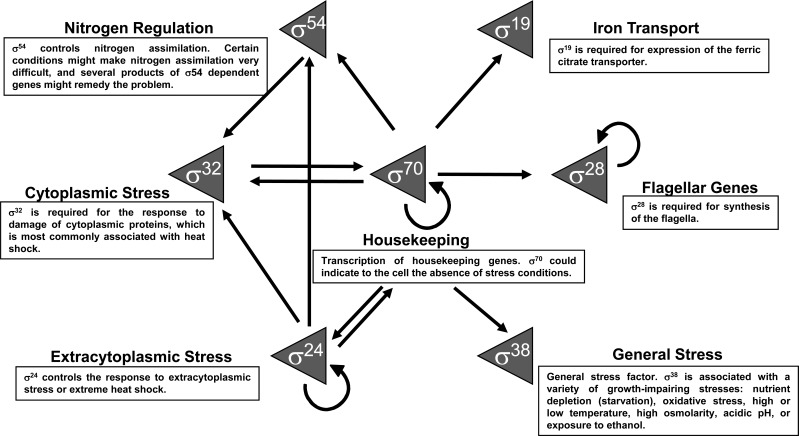
σ factors functions and their relationships at the transcriptional level. The arrows indicate the transcription interactions between σ factors.

In *Escherichia coli*, the vast majority of transcriptional “housekeeping” is accomplished by the RNAP core in complex with σ^70^. However, six other σ factors provide selectivity for transcribing different sets of genes. These genes, required for adaptive responses, have related functions and are transcribed in response to specific stimuli (Fig. **1**): σ^S^ (σ^38^) is the general stress factor associated with stationary phase and a variety of growth-impairing stresses; σ^H^ (σ^32^) controls heat shock responsive promoters; σ^F^ (σ^28^) is used for flagellum-related functions; σ^E^ (σ^24^) controls responses to extracytoplasmic or extreme heat shock stress; σ^FecI^ (σ^19^) is required in iron transport for transcription of the ferric citrate transporter; and σ^N^ (σ^54^) controls promoters for nitrogen assimilation. This repertoire of alternative σ factors reflects the lifestyle of *E. coli *as a gut commensal bacterium [2].

In growing *E. coli* cells, the number of RNAP holoenzymes involved in transcription is 1300 molecules per cell. There are also other 700 molecules per cell as free RNAP that has to be bound to any σ factor but is not involved in transcription [3]. Therefore, total RNAP is a limited resource. When a RNAP holoenzyme finishes the transcription of a given transcriptional unit, the σ factor dissociates from the RNAP core, and then other σ factors are able to compete for binding to RNAP core. The new RNAP holoenzyme incorporates to the pool of free RNAP [3]. Moreover, while RNAP core is not able to selectively bind to any σ factor, different σ factors have different affinities for RNAP core. As a consequence, the concentration of alternate σ factors requires strict regulation in order to properly control the delicate interplay favoring the competence for the scarce RNAP core. The concentration of σ factors is partially controlled by two types of protein-protein interaction regulatory elements: 1) anti-σ factors, which bind to and inhibit their cognate σ factor, and 2) adaptor proteins, which bind to their cognate σ factor and proteolyzed it. From a systems-level viewpoint, despite their mechanistic differences both are mechanisms of protein sequestration that have the same regulatory effect of removing the active σ factor from the cytoplasm, allowing the reprogramming of the basal transcriptional machinery towards a different set of target genes. Thus, for pragmatic reasons and taking an integrative approach, both regulatory elements will be called anti-σ factors in this review. Recently, it has been demonstrated that protein sequestration potentially can elicits an ultrasensitive response with a high apparent Hill-coefficient contrary to a traditional graded response [4]. In the context of the interaction between σ factors and their cognate anti-σ factors, this ultrasensitive response could enable an all-or-none response that drastically alters global gene expression. Moreover, the presence of feedback circuits enables the robust adaptability via a homeostatic response of *E. coli* to sudden environmental changes [1].

Since the great French scientist Claude Bernard (1813-1878), founder of modern experimental physiology, considered the stability of the *miliéu interieur* (internal environment), homeostasis mediated by feedback has been confirmed as one of the fundamental and ubiquitous mechanisms underlying life. One hundred years after Bernard’s seminal idea, cybernetics was developed by Norbert Wiener through formalizing the concept of feedback and mathematically demonstrating that negative feedback is a necessary condition to give rise to a teleological (goal-based) behavior in any system. In this control scheme, robust adaptation is created by feeding the output back into the input of the system, enabling it to respond to minimal deviations from the goal [5, 6]. This principle can be found not only in any man-made device that must maintain an internal steady state according to a design, from cisterns to electronic devices, but also in biological systems to allow robust internal stability under changing conditions [7]. A delicate balance exists between fragility and robustness in feedback control circuits [6]. The robustness of the final steady state (i.e., the return to the output value existing before perturbation) in response to a stimulus, such as heat shock, will arise at the price of a delicate or highly susceptible response in the dynamics of the circuit when a deviation from the steady state is sensed [7].

Molecular studies have determined specific roles for products of individual genes, but genomics has shown the decline of the traditional roles that have been assigned to proteins, such as σ factors and regulatory proteins. Despite their different regulatory mechanisms both proteins exerttranscriptional control in terms of their causal effects and both are important to the understanding of the regulatory hierarchy governing a bacterium [8]. From the perspective of systems biology, research has moved from individual genes to the regulation of systems. Although there have been a number of excellent studies on each of these σ/anti-σ post-transcriptional regulation systems [9-16], no comprehensive comparison of these mechanisms has been conducted in a single model organism. Certainly, regulatory proteins, σ factors, anti-σ factors, cofactor metabolites, and sRNAs, among other factors, shape a multi-layered complex regulatory network whose global understanding is a current challenge for systems biology. Both σ factors and anti-σ factors form one of these layers. Therefore, in this review we analyzed two issues from an integrative perspective: 1) the differences between the cellular components controlling the expression and function of σ factors and 2) the common principles governing σ/anti-σ post-transcriptional control in *E. coli*.

## ANTI-σ REGULATION

Adequately orchestrated gene expression is necessary for the successful adaptation and survival of cells. A strategy in bacteria to control gene expression is to displace the housekeeping σ subunit of the RNAP and replace it with an alternative σ factor. Then, with the new σ, the RNAP holoenzyme, can recognize and initiate transcription for a different type of promoter. Consequently, genes not being expressed can be transcribed drastically reprogramming global gene expression and enabling a proper response to an environmental cue [17]. To accomplish this, the activity of σ factors is mainly determined by both their intracellular levels and the activity of proteins called anti-σ factors. The latter binds to their cognate σ factor, blocking the formation of the transcription complex in the presence of a certain environmental cue [18]. This avoids the transcription of unnecessary genes in the new condition. Anti-σ factors regulate diverse processes including the stress response, flagellar biosynthesis, and enzymatic modification [19]. There are two classes of anti-σ factors: 1) the cytoplasmic anti-σ factors, and 2) the inner-membrane-bound anti-σ factors, also known as extra-cytoplasmic function (ECF) anti-σ factors [20]. The first class includes Rsd, HscC, DnaK, RssB, and FlgM and the second class contains RseA and FecR. Interestingly, all the anti-σ factors in *E. coli* are transcribed by the σ factor that they repress, creating a negative feedback circuit between the σ factor and its corresponding anti-σ.

## REGULATION OF σ^70^ BY Rsd AND HscC

The transcription initiation of genes in *E. coli* required for growth under optimum conditions is ensured by the σ^70^ factor. σ^70^ has two anti-σ factors, Rsd and HscC, which disable σ^70^-dependent transcription at the onset of stationary phase and also possibly heat shock, respectively. The latter is suggested by *in silico* evidence [21], so further research is needed to validate this possibility.

The transcriptional control of *rsd* has been validated *in vitro* [22]. It is transcribed from two promoters, P1 and P2, which are dependent on σ^38^ and σ^70^, respectively. In the promoter region of *hscC* two promoters, σ^70^ and σ^32^, have been proposed by *in silico* methods [21]. For graphical schemes of the organization and genomic context of genes coding for σ factors and anti-σ factors here discussed the reader is referred to (Supplementary Fig. **1**).

In stationary phase, the putative anti-σ^70^ factor Rsd binds to the region 4 helix-turn-helix motif of σ^70^, which is responsible for -35 promoter recognition, blocking association with the RNAP core [17]. This also contributes to the preferential use of σ^38^, which is the σ factor used upon entry into stationary phase [17, 19, 20, 23-32]. Reminiscent of Rsd, other *E. coli* anti-σ factors (i.e., FlgM, RseA, FecR, DnaK and RssB) also bind to multiple regions of their cognate σ factors to prevent the holoenzyme formation [33].

**Fig. (2):**
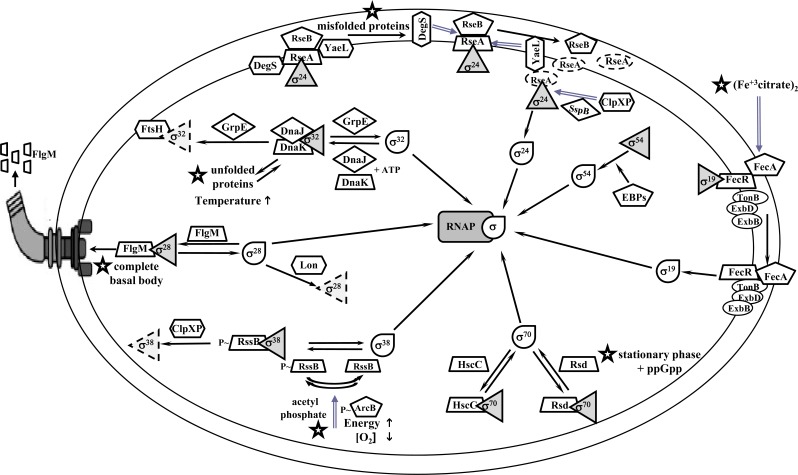
Regulation of σ factors by anti-σ factors in *E. coli*. The forms in figure represent: tear (σ factor active form), solid triangle (σ factor inactive form), dashed triangle (σ factor degraded form), star (environmental signal that releases the σ factor), trapezoid (anti-σ factor), pentagon (anti-σ factor sensor or modulator), solid ellipse (transducer signal complex), hexagon (protease), rhombus (chaperone), dashed ellipse (any protein in degraded form), rounded rectangle (RNAP core).

In contrast, HscC forms a complex with σ^70^ and may function as a negative modulator of housekeeping gene expression. This anti-σ is highly homologous to DnaK and HscA [34], two members of the Hsp70 chaperone family proteins in *E. coli*. The overexpression of *hscC* causes severe inhibition of cell growth. Primer extension and beta-galactosidase assays have shown that overexpression of HscC reduces σ^70^-dependent promoter activity. An *in vitro* transcription assay revealed that HscC inhibits σ^70^-dependent transcription of the *groESL* promoter. In addition, co-purification analysis showed that σ^70^ co-eluted with HscC [34]. HscC possesses the typical structural features of an Hsp70 protein, such as an ATPase domain, a substrate-binding domain, and a C-terminal domain [34]. However, rather than the substrate-binding domain, the ATPase domain of HscC contributes to its functional specificity for σ^70^, as suggested by the fact that chimeric proteins carrying the ATPase domain of HscC combined with other domains of either HscC or DnaK form complexes with σ^70 ^[34]. Promoter mutation analyses with wild-type and σ^38^ mutant backgrounds have suggested that P2 is the primary promoter for the transcription of *rsd* [35]. However, the contribution of σ^38 ^to P1 expression appears to be limited to a short period during the transition from exponential growth to stationary phase, with a maximal relative Rsd intracellular level that is only 20% of the σ^70^ level [36, 37]. Another report proposes that the levels of both Rsd and σ^70^ molecules in *E. coli *during stationary phase are similar, and that their association is significantly higher [33]. The mutation or deletion of Rsd did not reduce growth or alter the transcript levels of *E. coli* during stationary phase [37, 38]. Nevertheless, Rsd overexpression or a σ^70 ^highly affine Rsd mutant resulted in the up-regulation of several genes transcribed by σ^38^, indicating that Rsd enables the redistribution of RNAP to σ^38^-specific promoters by sequestering σ^70^ [38]. These observations suggest that the main function of Rsd is to promote σ^38 ^transcription. In fact, the transcription mediated by other alternative σ factors, such as σ^24^, σ^32^ and σ^54^, was also favored by Rsd overexpression [37-41]. Future studies are thus likely to focus on how the Rsd-binding activity is controlled in the exponential and stationary phases [35]. Although *rsd* transcription is not initiated primarily by σ^70 ^but also by σ^38^, the fact that σ^38^ is only transcribed by σ^70^ allows the formation of an indirect negative feedback circuit between σ^70^ and Rsd that could contribute to its regulation, which supports cellular homeostasis (Fig. **2**).

## REGULATION OF σ^38^ BY RssB

The σ^38^ factor is the master regulator of the general stress response, transcribing more than 140 genes that confer resistance against adverse conditions, such as stationary phase, oxidative stress, UV radiation, heat shock, hyper-osmolality, acidic pH, and ethanol [23].

In evolutionary terms, this σ factor is closely related to σ^70^ and therefore does not have a full unique consensus sequence. Specific promoter selectivity is achieved by a broader tolerance for deviations from 1) the optimal promoter sequence, or 2) the exact geometric alignment of the -35 and -10 regions requiring an optimal spacer length of 17 bp [42]. Additionally, trans-acting regulatory factors, such as regulatory proteins or regulatory RNAs [24], that bind to a promoter region contribute to RNAP holoenzyme selectivity. However, the cellular levels of σ^38^ are believed to reach a maximum of one-third of those of σ^70 ^[22, 25]. Given all these in addition to the lower affinity of σ^38^ for the RNAP core [3, 18, 28-31, 43], mechanisms ensuring that σ^38^ can recruit enough RNAP cores to carry out its transcriptional program are needed.

RssB (also named SprE) is a response regulator that functions as an anti-σ factor adaptor and plays a critical role in the control of cellular σ^38^ levels in *E. coli*. An *rssB* mutant has near constitutively high levels of σ^38^ but is impaired in the post-transcriptional regulation of σ^38 ^[44]. Two promoters transcribe *rssB*: the first, P1, is a distal promoter transcribed by σ^38^, which also co-transcribes *rssA*, and the second, P2, is situated in the *rssA-rssB* intergenic region from which *rssB* is transcribed in a σ^38^-dependent fashion [45].

The proteolysis of σ^38^ depends on three factors: the ClpXP protease, RssB, and a “turnover element”. The interface with RssB is the lysine-173 of the σ^38^ amino acid sequence. Binding of RssB to σ^38^ is stimulated by phosphorylation of the RssB receiver domain, suggesting that environmental stress affects σ^38^ proteolysis by modulating RssB affinity for σ^38 ^[46].

Therefore, the cellular levels of σ^38^ are regulated at different points (transcription, translation, and proteolysis) by RssB and the two-component system ArcB/ArcA in a "three-component system" that coordinates *rpoS* transcription and σ^38^ proteolysis and, thereby, maintains low σ^38^ levels in cells growing exponentially. In this system, it has been suggested that the sensor histidine kinase ArcB monitors both the oxygen and energy supplies [47]. In addition, this histidine-kinase phosphorylates ArcA (their cognate two-component transcriptional repressor regulator for RpoS) and RssB (a factor targeting σ^38 ^for proteolysis) [47]. Both phosphorylated ArcA and RssB keep σ^38^ levels low and are limiting factors in σ^38 ^proteolysis. Acetyl phosphate is an accessory component that contributes to RssB and, possibly, ArcA phosphorylation [48]. ClpXP is a protease that requires the exposure of σ^38^ to phosphorylate RssB [46, 49]. Given that σ^38 ^is protected against degradation when it is bound to the RNAP core, its concentration affects its own rate of degradation [47]. Once the cell enters in stationary phase or some other stress conditions are present, σ^38^concentration is increased above that of non-phosphorylated RssB, thus allowing σ^38 ^to bind to the RNAP core and transcribe its target genes [46]. In this case, the negative feedback circuit is formed directly between σ^38^ and RssB because the first factor transcribes the second, assuring the production of the anti-σ RssB and the control of σ^38^ when the environmental stress that triggered its liberation disappears (Fig. **2**).

## REGULATION OF σ^32^ BY DnaK

A temperature of 37°C or higher compromises *E. coli *homeostasis fundamentally due to thermal denaturation of folded proteins. In reaction to an upshift in temperature, *E. coli* employs the heat shock response to increase the activity of transcription directed by σ^32^ factor, inducing the up-regulation of over 120 products of this sigmulon. Some proteins produced under heat-shock conditions act as chaperones, such as GroEL and DnaK, which prevent protein turnover by maintaining proper folding, and proteases, such as Lon and Clp, which facilitate protein turnover (Fig. **2**).

The *dnaK* gene is co-transcribed with *dnaJ* from three heat-inducible promoters, which are recognized *in vitro* by a RNAP containing σ^32^ but not by a RNAP in complex with σ^70 ^[50]. An intracellular balance of molecular concentrations of chaperones in response to environmental conditions has significance in cellular homeostasis. The overexpression of *dnaK* does not have a deleterious effect on the chaperone system function, cell growth, survival and morphology [51], and also confers freeze tolerance [52].

σ^32^, the σ factor responsible for responding to heat shock, is maintained at low concentrations during exponential cell growth [53]. During optimal growth conditions, DnaJ (Hsp40 homolog) binds to σ^32^ (in the presence of ATP), presenting it to the ATP-bound DnaK (Hsp70 homolog). In this way, DnaK competes with the RNAP core to bind σ^32^. The complex of DnaJ-σ^32^-DnaK-ADP is formed after ATP hydrolysis. Then, the chaperone GrpE presents σ^32 ^to the free FtsH protease for degradation [19, 54]. Under stress conditions, cytoplasmic proteins become denatured, acting as substrates for the DnaJ/DnaK/GrpE chaperones complex, releasing active σ^32^ and allowing the transcription of heat shock genes [30, 55]. This free σ^32 ^can re-associate with the RNAP core to transcribe σ^32^ dependent promoters. As for σ^38^, σ^32^also takes part in the transcription of the components of its anti-σ system, ensuring that it is repressed when the environmental condition inducing its liberation (i.e., any condition responsible for cytoplasmic misfolded proteins) disappears (Fig. **2**).

## REGULATION OF σ^28^ BY FlgM

The flagellar genes of *E. coli* are organized into a regulatory hierarchy involving three classes of promoters, which groups genes according to their corresponding hierarchical level: early, middle and late. These classes of promoters are differentially expressed just prior the flagellum assembly in discrete intervals of the cell cycle.

σ^28^ activates the late genes involved in flagellum assembly. σ^28^ is initially expressed by an FlhDC-controlled class 2 promoter and is responsible for class 3 transcription of the flagellar regulon. In this stage, the proteins composing the flagellar filament, motility control and chemotaxis are expressed. In the late transcription hierarchical class 3, σ^28^ expression is also increased by transcription at an additional class 3 promoter [56, 57]. In contrast, the anti-σ gene *flgM* is transcribed from two promoters. The P1 promoter, which is regulated by σ^70^, is situated in the *flgA-flgB* intergenic region, and *flgM *is co-transcribed with *flgA* and *flgN*. The second promoter, P2, is situated upstream of *flgM* and is transcribed in a σ^28^-dependent fashion [57].

To avoid the participation of σ^28^ during assembly of the basal body of the flagellum, σ^28^activity needs to be inhibited. This process is carried out by the anti-σ factor FlgM [58]. In this manner, FlgM inhibits the σ^28^-dependent transcription of genes whose products are needed late in assembly, when the flagellar basal motor structure, the hook-basal body (HBB), is constructed [59]. With the assembly of the HBB, a type III secretion system is formed that is capable of secreting both the flagellar filament subunits and, interestingly, FlgM. This constitutes the other function of σ^28^, which facilitates the secretion of FlgM through the HBB, acting as an FlgM type III secretion chaperone. This results in the release of σ^28^, which allows σ^28^ to bind to a RNAP core and initiate transcription of the structural class 3 genes necessary to complete the flagellum [59].

Free σ^28 ^is a proteolysis substrate just like other σ factors. The Lon protease is responsible for σ^28^ proteolysis. When σ^28^ is in excess, its proteolysis increases the robustness and precision of this complex control system by rapidly reestablishing a 1:1 stoichiometry between σ^28^ and FlgM. This contributes to limiting the up-regulation of flagellar biosynthesis, and FlgM has a protective function against σ^28^ degradation [60]. When the flagellar filament is assembled completely, the anti-σ FlgM produced from the transcription regulated by σ^28 ^increases and then sequesters σ^28^ to suppress its function and proteolysis, forming a negative feedback regulatory circuit (Fig. **2**).

## REGULATION OF σ^24^ BY RseA

Under certain stresses (e.g., heat or osmotic shock) misfolded envelope proteins are generated. As a consequence, bacteria such as *E. coli* have developed signaling pathways that sense and respond to these envelope stresses. These pathways trigger the expression of genes encoding a wide variety of proteins that are able to repair envelope (e.g., periplasmic folding chaperons, proteases, and biosynthesis enzymes for lipid A). These pathways are known as extracytoplasmic (EC) or envelope stress responses. One of the EC systems monitoring envelope stress is the σ^24^ sigmulon, where σ factor is induced when the stress damages the periplasmic proteins. This is the only known EC response that is essential for viability in *E. coli* K-12 [61], whose misfolded-proteins in the cell envelope direct the expression of genes to restore envelope integrity [62].

The EC stress signal is transduced across the inner membrane to the cytoplasm via the inner membrane protein RseA, which is the anti-σ factor responsible for inhibiting σ^24^, and it is also known as an EC anti-σ factor. In general, the EC anti-σ factors are inner membrane proteins with at least one transmembrane domain. Additionally, it has been observed that the cytoplasmic N-terminal domain of EC anti-σ factors is conserved and possibly interacts directly with their cognate σ factor. On the other hand, little homology is observed in their transmembrane C-terminal domains (except between *E. coli *RseA and *H. influenzae* MclA), which is consistent with the possibility that these anti-σ factors may respond to different extracytoplasmic signals [20].

The *rseA* gene, which encodes the anti-σ^24 ^factor, is transcribed from three promoters. Two of these promoters, P1 and P2, are located upstream the gene *rpoE*; while the third promoter, P3, is located inside the coding region of *rpoE*. No obvious σ^70^ consensus promoter sequence is found upstream of promoter P1 [63]. However, promoters P2 and P3 have the traditional conserved motifs sequences of σ^24^-dependent transcription regulation [64]. Therefore, the levels of σ^24^ factor partially depend on the levels of its cognate anti-σ factor and vice-versa.

RseA anchors to the inner membrane (single pass) and contains a binding site for σ^24^. Under unstressed conditions, biochemical analyses and crystallographic structures have shown that the periplasmic domain of RseA interacts with RseB (protecting RseA from proteolysis), while the RseA cytoplasmic domain is sandwiched between two domains of σ^24^. This interaction occludes the two main binding regions of σ^24^ for the RNAP core, repressing σ^24^ activity because it is sequestered at the inner membrane by the anti-σ factor RseA [65].

During envelope stress, which interferes with the folding of outer membrane proteins (OMPs) and includes heat shock, overexpression of OMP genes, and mutations in genes encoding the chaperones required for OMPs folding, *E. coli* employs a proteolytic cascade to transduce signals related to protein folding in the periplasm to the transcriptional apparatus in the cytoplasm. RseA is degraded in response to misfolded OMPs by the sequential action of two inner membrane proteases, DegS and YaeL (RseP). The C-termini of misfolded OMPs bind to the PDZ domains (a common structural domain found in cellular signaling proteins) of the trimeric DegS protease, activating cleavage of the periplasmic domain of RseA [66, 67]. The partially degraded RseA is now a substrate for YaeL. YaeL cleaves RseA further, releasing the cytoplasmic domain of RseA bound to σ^24^. The cascade activates the cytoplasmic ClpXP protease and SspB chaperone, which degrades the remaining domain of RseA in the final step of the proteolytic cascade, thus allowing the release of σ^24 ^[68, 69]. Then, σ^24^ binds to the RNAP core and is ready to direct transcription of its sigmulon to cope with the environmental condition produced by the envelope stress [70]. The regulatory nucleotide ppGpp, which mediates the stringent response, and the RNAP-binding transcription protein DksA together activate σ^24^-dependent transcription, once σ^24^ is released by RseA, through subverting the dominant housekeeping σ^70^. In fact, σ^24^ activity is induced upon entry into stationary phase in *E. coli* by the alarmone ppGpp and not via the RseA-dependent stress signaling pathway [71]. When periplasmic stress disappears, the σ^24^-RseA complex is restored by the RseA transcription for σ^24^, which replaces the anti-σ RseA that was degraded by proteolysis (Fig. **2**).

## REGULATION OF σ^19^ BY FecR

The other EC signaling pathway is the σ^19 ^(FecI) sigmulon of *E. coli*. Two signals are necessary to activate the expression of *fecI*: iron starvation and the presence of ferric-citrate in the environment. Whereas iron starvation is sensed by the Fur protein, iron citrate is bound by the FecA receptor in the outer membrane [72].

A σ^19^ deletion mutation is totally devoid of ferric citrate transport system expression because expression of *fecIR* mRNA requires σ^70^ and the transcription of the *fecABCDE* transport genes is regulated by σ^19^, which responds to ferric citrate via the anti-σ system FecA-FecR. *fecI* and *fecR* co-transcription is inhibited by the iron-loaded Fur repressor, which in turn transcribes the *fec* transport genes at a low level [73].

Ferric citrate serves as an iron source for *E. coli *[74]; however, the transport of this ferric chelator into the periplasm requires energy and a functional complex of three inner membrane proteins: TonB, ExbB, and ExbD. A physical interaction between the periplasmic portion of TonB and FecA results in the transduction of energy from the proton gradient across the inner membrane [72, 75]. This energetic cost may be responsible for the low position of ferric citrate in the hierarchy of iron sources.

FecA (an outer membrane protein), which recognizes ferric citrate, serves a dual role: It is 1) an iron transporter of ferric citrate across the outer membrane that operates with the *fec* transport gene operon, and 2) an anti-σ sensor that regulates the transcription of the ferric citrate transport genes *fecABCDE* in the cytoplasm. Both processes are independent and require energy transduction by the inner membrane TonB-ExbB-ExbD complex [75].

FecA, the anti-σ sensor, elicits a signaling cascade from the cell surface to the cytoplasm [29]. This signaling flux involves a series of conformational changes of the proteins FecA (anti-σ sensor), FecR (anti-σ factor), and FecI (EC σ factor), respectively.

It has been proposed that σ^19^ binds to its anti-σ factor, FecR; thus, FecR sequesters σ^19^ until FecR is inactivated by undergoing a structural change in responseto the induction signal of FecA (iron-citrate).

Activated FecA interacts in the periplasmic space with the C-terminus of FecR (anchored in the cytoplasmic membrane). Then, the signal is transferred across the cytoplasmic membrane into the cytoplasm via FecR N-terminus [76]. Thus, FecR releases the σ^19^ factor, allowing activation of the σ^19^ factor that directs the RNAP core to induce transcription of the *fecABCDE* transport genes [77]. σ^19^ contributes to the transcription of the anti-σ sensor FecA. When ferric citrate becomes exhausted or a different iron source becomes available, FecR is reverted to its prior conformation by FecA, and FecR becomes able to sequester σ^19 ^thus inhibiting the transcription of its corresponding genes (Fig. **2**).

## DOES σ^54 ^HAVE AN ANTI-σ FACTOR?

The second family of σ factors in *E. coli* is exclusively represented by σ^54^, which directs the recognition of distinct promoter motifs located at positions -24 and -12 relative to the transcriptional start site. Although initially identified for their role in nitrogen assimilation, σ^54 ^is utilized in different physiological processes and can remedy certain conditions that make nitrogen assimilation very difficult [78]. σ^54^ does not have primary sequence similarity to σ^70^ proteins and regulates transcription by a different mechanism. σ^54^ can not spontaneously isomerize (melt) DNA to form open promoter complexes. This step of transcription strictly depends on mechanotranscriptional activators (also known as bacterial enhancer binding proteins, or bEBPs) that utilize ATP hydrolysis to drive conformational changes for this transition [79]. Twelve bEBPs have been described in *E. coli* [28, 78, 79]. Apparently, σ^54^ factor does not have an anti-σ factor. The σ^54 ^impairing to transcribe genes without the help of σ^54^-dependent transcriptional activators (bEBPs) may explain the ostensible absence of an anti-σ factor.

## SYSTEMS-LEVEL LESSONS FROM σ/ANTI-σ CIRCUITS

Biological systems are, both intrinsically and extrinsically, noisy. Therefore, a mechanism enabling the cell to maintain a stable internal state is required. For maintain homeostasis, organisms need to detected changes in their environment and respond accordingly in a variety of manners. Negative feedback circuits have evolved in organisms as a common tool for maintaining homeostasis and filtering noise, thus providing a robust response. A feedback mechanism occurs when an organism senses an internal or external environmental perturbation and changes the level or activity of a substance, thus starting a regulatory cascade that eventually reverses the initial perturbation. While negative feedback circuits are ubiquitous in biological regulation, certain design principles emerge as lessons from the evolution of feedback in the anti-σ control.

**Fig. (3):**
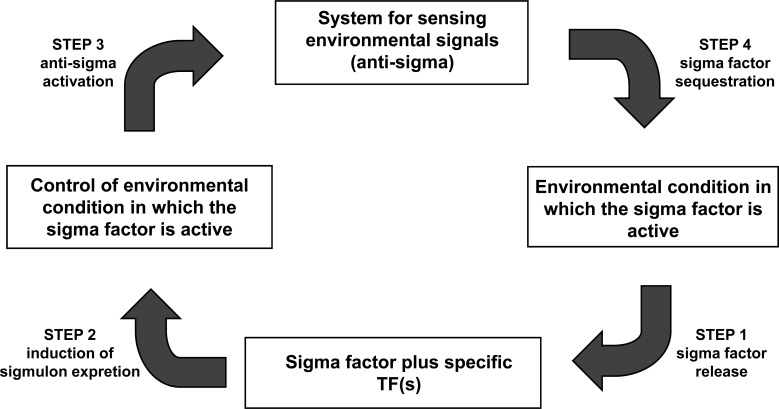
Conditions needed to be fulfilled to generate a negative feedback circuit of σ/anti-σ factors assuring an efficient and robust cellular response.

Through this review, we surveyed the different regulatory systems formed by the σ and anti-σ factors in *E. coli*. Following an integrative approach, we were not only interested in describing their biological differences, but also their systems-level similarities. (Fig. **2**) shows the intricate cellular complexity of the σ/anti-σ circuitry. Despite this complexity, a small set of common mechanisms could be outlined. First, the anti-σ factor releases its cognate σ factor in response to a specific environmental signal (step 1, Fig. **3**). It has been reported that the latter causes a rapid increase in the cognate σ factor concentration [1-3], supporting the ultrasensitive response discussed in the Introduction. Second, the σ factor and other transcriptional factors participate, directly or indirectly, in the transcription of their correspondent anti-σ factor (step 2, Fig. **3**). Third, the anti-σ factor plays one of two different roles: 1) it is kept inactive by other factors or adaptors (antagonistically by anti-anti-σ factorsor anti-adaptors, and cooperatively by co-anti-σ factors, or post-translationally by proteolysis) during the presence of the specific environmental signal, or 2) it participates actively in the response to the environmental signal (step 3, Fig. **3**). Finally, when this signal disappears, anti-σ factors efficiently repress their σ factors by sequestration (step 4, Fig. **3**). The sequestered σ factor has two possible fates: it can be protected against proteolysis or proteolyzed. The negative feedback circuits generated by these conditions in all alternative σ factors (except σ^54^) and their cognate anti-σ factors interactions enable the cell to return to a stable condition similar to that before the environmental signal. An interesting exception to these principles is the housekeeping σ^70^ factor. As all the alternative σ factors, σ^70^ also transcribes its anti-σ factors (*rsd* and *hscC*). Nevertheless, there are some intriguing differences. Both anti-σ^70^ factors seem to be also transcribed by alternative σ factors. As previously discussed, the *rsd* expression has been validated *in vitro *to be promoted by σ^70^ and σ^38^, while the promoter region of *hscC *has two promoters, σ^70^ and σ^32^, which have been proposed *in silico*. Whether these predictions are functional requires further research. However, this evidence opens the possibility that the σ^70^ concentration may be indirectly controlled by entry into stationary phase and heat shock, respectively, two conditions requiring alternative σ factors. Whether these are the only environmental signals controlling the availability of σ^70^ remains an open question.

## CONCLUSIONS

Mechanisms for coordinating the regulation of gene expression are hierarchic and complex. This coordination appears to be critical for the maintenance of a balanced gene expression profile during various environmental conditions. This is achieved by two different but complementary mechanisms. On the one hand, transcriptional regulatory protein compete with many other regulators to redirect the RNAP to transcribe a set of genes. On the other hand, σ factors show a rapid increase in their concentration and then compete with a small number of other σ factors for the limited RNAP core. The scarce RNAP core is a key aspect for the successful functioning of this regulatory mechanism through competence. Then, by activating alternative σ factors, gene expression can be efficiently and globally reprogrammed to respond to sudden environmental cues. As a consequence, the specificity that σ factors confer to the RNAP for certain promoters allows the circumvention of the transcriptional regulatory proteins currently active in a previous growth condition enabling a specific and efficient response to the new condition.

The availability of *E. coli *σ factors is regulated at multiple levels by specific signal transduction pathways. These pathways are activated in response to specific stresses or environmental conditions. Several interactions play a prominent role in this regulatory process. These interactions operate synergistically and shape complex regulatory networks and cascades, constituting the regulatory mechanism establishing the gene expression profile. Thus, the cell can have a wide range of gene expression profiles that allow cells to cope with different requirements, such as sudden environmental changes or developmental programs. Anti-σ systems evolved in *E. coli* as a hierarchy of negative feedback interactions, allowing the cell to control the gene expression in response to specific environmental conditions in a way resembling compartmentalization by partitioning the gene expression states into a set of discrete functional units. When these environmental conditions disappear, the system relieves gene expression thereby completing the cycle.

To allow the efficient response of some σ factors, it is important that they are present in the cell at basal levels at all times. This may be achieved by σ^70^, whose promoter is present in the upstream region of each alternative σ factor (Fig. **1**). Nevertheless, σ activity has to be strictly controlled by preventing its binding to the RNAP core. This also have the desired effect of silencing the genes not required under a certain biological context. Anti-σ factors facilitate this requirement by operating as a system that senses the environment and sequesters the σ factor when the gene expression driven by them is superfluous given the biological context.

## SUPPLEMENTARY MATERIALS

Supplementary material is available on the publisher’s web site along with the published article.
