# Supplementary material for: Anti-Sigma Factors in E. coli: Common Regulatory Mechanisms Controlling Sigma Factors Availability
Source: Curr Genomics. 2013 Sep;14(6):378–87. doi: 10.2174/1389202911314060007 (PMC3861889; doi:10.2174/1389202911314060007)
Supplement: Supplementary file 1 [file CG-14-378_SD1.pdf]

Supplementary Material

rpoD **(σ^{70}) Gene Local Context**
Synonyms: *alt*, sigma D factor, sigma 70 factor, σ^{70}

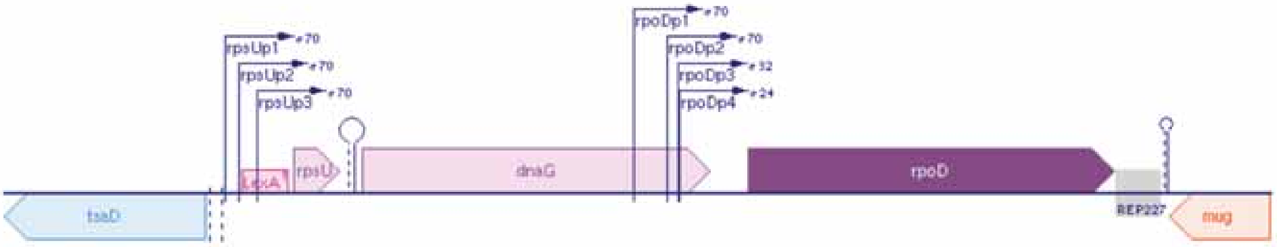

rsd **(Anti-σ^{70}) Gene Local Context**
Synonyms: *yjaE*

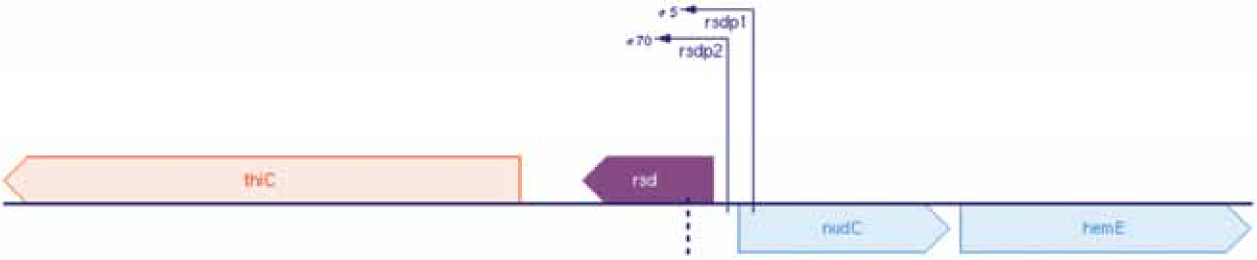

hscC **(Anti-σ^{70}) Gene Local Context**
Synonyms: *ybeW*, Hsc62

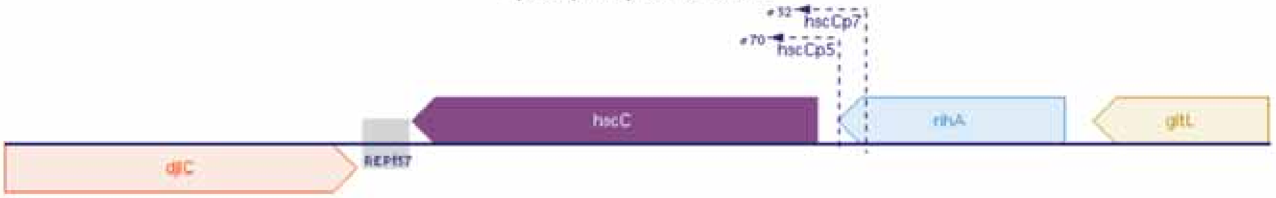

rpoS **(σ^{38}) Gene Local Context**
Synonyms: *sigS*, *otsX*, *abrD*, *appR*, *csi2*, *dpeB*, *katF*, *nur*, sigma 38 factor, sigma S factor, σ^S

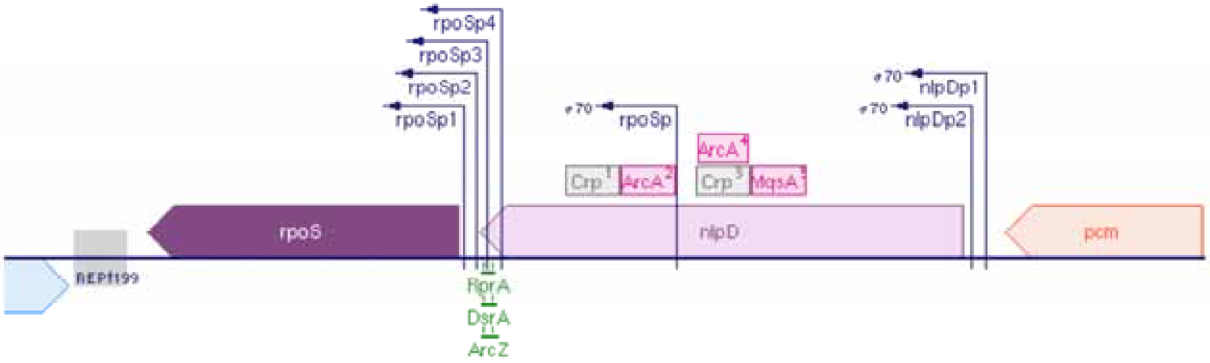

rssB **(Anti-σ^{38}) Gene Local Context**
Synonyms: *ychL*, *sprE*, *hnr*

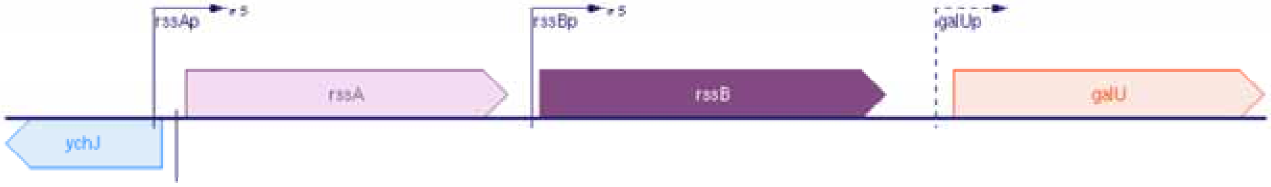

rpoH (σ^{32}) Gene Local Context

Synonyms: *fam*, *hin*, *htpR*, sigma H factor, sigma H, sigma 32, sigma 32 factor, σ^{32}

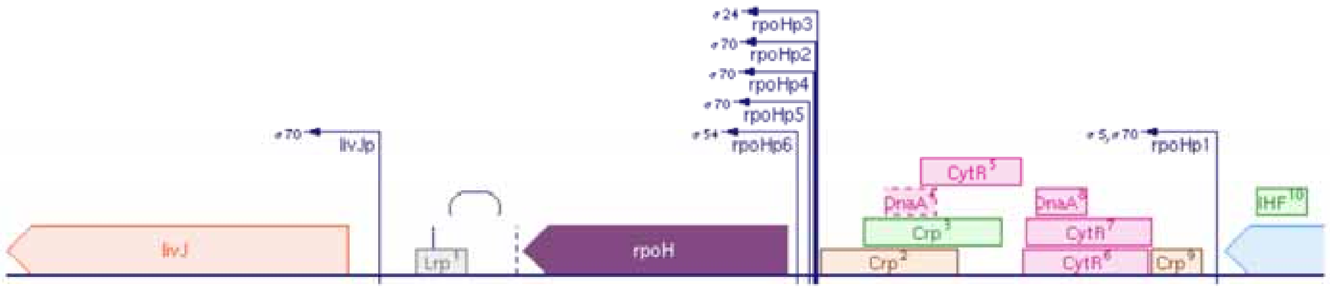

dnaK (Anti-σ^{32}) Gene Local Context

Synonyms: *groPF*, *groPC*, *seg*, *grpF*, *grpC*, *groPAB*, Hsp70 chaperone DnaK, heat shock protein 70kDa

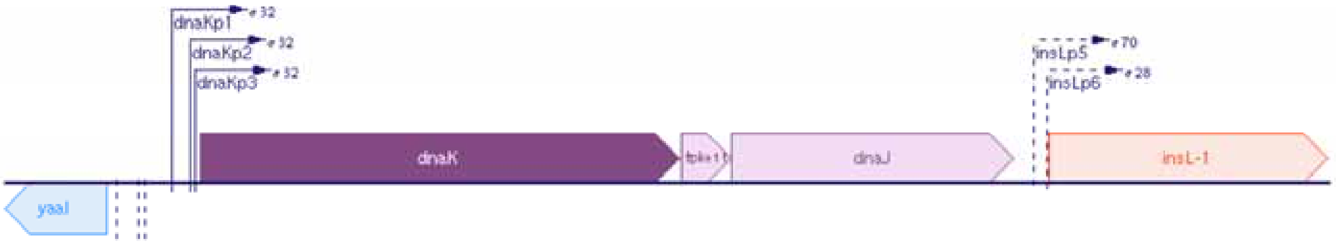

fliA (σ^{28}) Gene Local Context

Synonyms: *rpoF*, *flaD*, sigma F factor, sigma 28 factor, σ^{28}

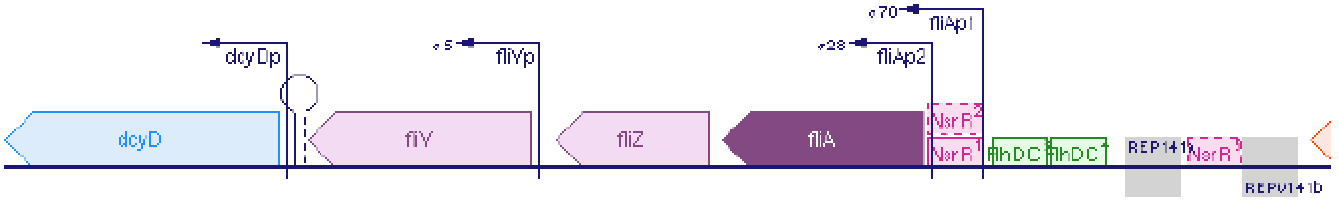

flgM (Anti-σ^{28}) Gene Local Context

Synonyms: none

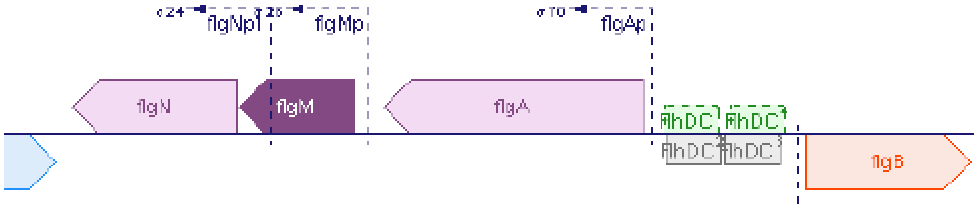

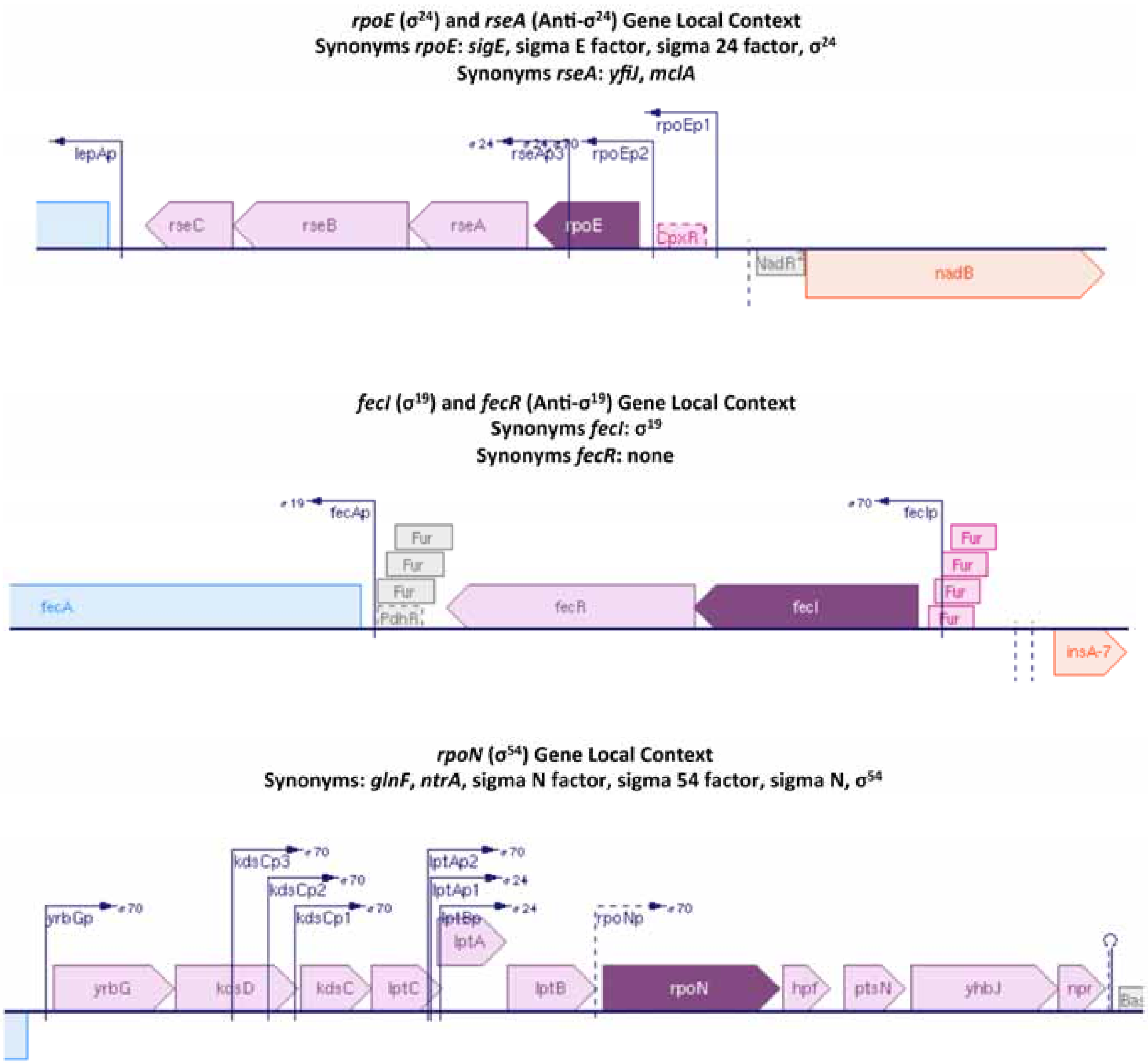

Supplementary Fig. (1). Graphical schemes of organization and the genomic context of genes coding for σ factors and anti-σ factors discussed in this review.Additional information of these genes can be found on the EcoCyc database (http://ecocyc.org/).
